# Supplementary material for: A Likelihood Approach for Real-Time Calibration of Stochastic Compartmental Epidemic Models
Source: PLoS Comput Biol. 2017 Jan 17;13(1):e1005257. doi: 10.1371/journal.pcbi.1005257 (PMC5240920; doi:10.1371/journal.pcbi.1005257)
Supplement: S1 File — (TAR.GZ) [file pcbi.1005257.s014.tar.gz › HSPH_Online-SI-Revision/Additions/EnKF08/n100-extreme_noisy/n100-extreme_noisy_EnKF2_table4.pdf]

1	newly 3 weeks spec	2278.3	3578.26	{95., 16 515.}	166.5	0.718758	newly 3 weeks cumul	9885.83	14 895.4	{626., 73 326.}	154.198	0.634245	newly 5 weeks spec	1200.81	1467.01	{46., 5285.}	141.027	0.777061	newly 5 weeks cumul	12 674.4	18 673.6	{722., 87 385.}	153.499	0.650377	newly 50 weeks cumul	18 298.5	21 190.1	{722., 92 673.}	133.786	0.709561
2	newly 3 weeks spec	2435.	5255.65	{157., 22 554.}	194.126	0.619471	newly 3 weeks cumul	9225.	35 933.5	{8953., 87 724.}	299.458	0.480349	newly 5 weeks spec	1337.	1453.1	{0., 6258.}	126.767	1.43252	newly 5 weeks cumul	12 421.	40 221.1	{9896., 106 494.}	236.153	0.413237	newly 46 weeks cumul	16 331.	41 698.5	{11 519., 112 314.}	177.248	0.367263
3	newly 3 weeks spec	1444.	2068.95	{81., 6072.}	163.679	0.766453	newly 3 weeks cumul	6755.	5993.79	{679., 17 691.}	94.7409	0.553651	newly 5 weeks spec	609.	2194.83	{8., 11 479.}	386.284	1.09612	newly 5 weeks cumul	8307.	10 457.1	{781., 35 313.}	133.152	0.611168	newly 51 weeks cumul	9551.	17 867.6	{813., 81 759.}	192.508	0.681301
4	newly 3 weeks spec	766.	2883.18	{0., 8129.}	339.856	1.04136	newly 3 weeks cumul	4576.	14 175.4	{1095., 41 412.}	250.643	0.417978	newly 5 weeks spec	254.	1594.92	{0., 3400.}	596.654	1.05638	newly 5 weeks cumul	5299.	17 929.8	{1169., 49 662.}	279.619	0.441443	newly 55 weeks cumul	5634.	21 430.3	{1196., 79 933.}	321.528	0.467798
5	newly 3 weeks spec	1978.	1292.29	{168., 3287.}	81.5445	0.593997	newly 3 weeks cumul	9313.	7021.77	{1588., 16 387.}	80.4631	0.489471	newly 5 weeks spec	894.	460.4	{19., 1459.}	80.7517	0.768226	newly 5 weeks cumul	11 542.	8239.61	{1813., 19 526.}	78.9817	0.509174	newly 52 weeks cumul	13 327.	9073.28	{1967., 21 620.}	78.2006	0.531469
6	newly 3 weeks spec	2370.	712.75	{0., 1062.}	96.8156	2.60334	newly 3 weeks cumul	9890.	17 445.2	{1400., 89 820.}	173.784	0.513227	newly 5 weeks spec	1132.	293.63	{0., 621.}	100.884	2.662	newly 5 weeks cumul	12 727.	18 150.4	{1503., 91 177.}	151.194	0.55977	newly 50 weeks cumul	15 600.	19 591.2	{1547., 91 177.}	139.697	0.59746
7	newly 3 weeks spec	1951.	2540.54	{0., 2516.}	135.877	1.05462	newly 3 weeks cumul	8547.	19 713.1	{1130., 72 885.}	163.114	0.344165	newly 5 weeks spec	968.	930.11	{0., 1104.}	128.759	1.45758	newly 5 weeks cumul	10 776.	22 170.7	{1367., 88 150.}	148.056	0.349781	newly 48 weeks cumul	12 929.	23 665.7	{1659., 93 585.}	137.764	0.375114
8	newly 3 weeks spec	2287.	600.41	{0., 2485.}	88.8793	2.33397	newly 3 weeks cumul	9859.	10 059.6	{1368., 24 853.}	110.451	0.54495	newly 5 weeks spec	1178.	264.51	{0., 543.}	94.9601	2.58994	newly 5 weeks cumul	12 762.	10 676.4	{1559., 25 698.}	98.8386	0.588582	newly 50 weeks cumul	15 717.	12 077.	{1644., 26 857.}	96.3432	0.631985
9	newly 3 weeks spec	2292.	1580.94	{45., 4188.}	92.7906	0.847443	newly 3 weeks cumul	10 287.	5598.03	{635., 10 393.}	78.7968	0.692848	newly 5 weeks spec	1160.	1315.15	{2., 3755.}	138.892	1.20023	newly 5 weeks cumul	13 098.	8338.88	{789., 20 230.}	83.7546	0.720605	newly 50 weeks cumul	15 554.	18 724.9	{953., 69 678.}	138.605	0.827703
10	newly 3 weeks spec	296.	3208.18	{520., 9648.}	989.723	0.796948	newly 3 weeks cumul	1966.	13 827.2	{2702., 33 562.}	609.276	0.655589	newly 5 weeks spec	92.	1731.17	{103., 3375.}	1787.9	0.90865	newly 5 weeks cumul	2237.	17 930.	{3795., 62 475.}	707.474	0.698454	newly 54 weeks cumul	2337.	22 376.9	{3899., 75 202.}	863.433	0.755912
11	newly 3 weeks spec	2110.	2216.37	{0., 2974.}	148.411	2.1804	newly 3 weeks cumul	9055.	14 602.4	{1041., 27 045.}	145.948	0.53273	newly 5 weeks spec	1137.	1198.2	{0., 845.}	159.532	2.16085	newly 5 weeks cumul	11 827.	17 446.6	{1077., 31 139.}	141.132	0.563688	newly 48 weeks cumul	14 734.	20 370.4	{1086., 43 937.}	138.239	0.59014
12	newly 3 weeks spec	2018.	1042.7	{157., 2124.}	66.5164	0.542653	newly 3 weeks cumul	9115.	9194.18	{1623., 17 035.}	88.9611	0.442697	newly 5 weeks spec	959.	302.67	{19., 723.}	72.5224	0.838431	newly 5 weeks cumul	11 517.	10 028.	{1734., 17 472.}	78.8005	0.445158	newly 49 weeks cumul	13 641.	10 636.3	{1760., 17 606.}	73.3545	0.454114
13	newly 3 weeks spec	3118.	3114.25	{474., 6920.}	70.8977	0.398763	newly 3 weeks cumul	12 156.	27 840.8	{5990., 74 283.}	163.941	0.327113	newly 5 weeks spec	2008.	646.9	{55., 1004.}	81.4681	0.894732	newly 5 weeks cumul	16 586.	29 858.1	{6167., 80 901.}	129.459	0.342145	newly 47 weeks cumul	22 880.	30 523.	{6189., 87 594.}	101.382	0.372738
14	newly 3 weeks spec	249.	887.87	{149., 3445.}	277.715	0.362647	newly 3 weeks cumul	1934.	5092.08	{908., 13 292.}	187.807	0.333628	newly 5 weeks spec	37.	347.64	{26., 913.}	851.459	0.548704	newly 5 weeks cumul	2082.	5981.37	{1033., 18 093.}	210.083	0.345293	newly 52 weeks cumul	2131.	6695.98	{1083., 24 309.}	235.941	0.358004
15	newly 3 weeks spec	658.	1963.78	{135., 6788.}	254.483	0.46886	newly 3 weeks cumul	3705.	10 609.7	{1765., 33 447.}	220.106	0.358631	newly 5 weeks spec	197.	739.81	{18., 3284.}	347.843	0.627257	newly 5 weeks cumul	4246.	12 543.7	{1793., 39 651.}	232.531	0.38161	newly 52 weeks cumul	4525.	13 879.8	{1796., 44 007.}	245.817	0.402285
16	newly 3 weeks spec	902.	730.06	{80., 1434.}	65.3459	0.494677	newly 3 weeks cumul	5020.	18 571.2	{3111., 59 629.}	289.355	0.456181	newly 5 weeks spec	342.	64.2	{2., 120.}	88.2807	1.35058	newly 5 weeks cumul	5877.	18 829.9	{3116., 59 853.}	244.048	0.428444	newly 52 weeks cumul	6347.	18 872.2	{3116., 59 860.}	222.926	0.413185
17	newly 3 weeks spec	2633.	3174.13	{403., 7353.}	91.9464	0.455573	newly 3 weeks cumul	10 424.	19 130.6	{4860., 47 197.}	126.797	0.347852	newly 5 weeks spec	1461.	786.56	{59., 1521.}	70.1752	0.667126	newly 5 weeks cumul	13 884.	21 515.4	{5052., 59 464.}	108.865	0.368818	newly 47 weeks cumul	18 108.	22 368.2	{5081., 62 852.}	90.3267	0.392859
18	newly 3 weeks spec	1965.	2092.56	{0., 3228.}	133.248	2.0662	newly 3 weeks cumul	8760.	23 806.3	{1733., 87 324.}	236.388	0.5052	newly 5 weeks spec	847.	872.49	{0., 1392.}	123.973	2.02973	newly 5 weeks cumul	10 903.	25 998.	{2442., 92 961.}	205.09	0.49256	newly 48 weeks cumul	12 657.	29 097.6	{2952., 95 804.}	185.477	0.447676
19	newly 3 weeks spec	2260.	1464.93	{0., 1350.}	124.228	2.58172	newly 3 weeks cumul	9867.	14 832.1	{1859., 48 498.}	138.118	0.464966	newly 5 weeks spec	1026.	1020.81	{0., 953.}	146.726	2.38534	newly 5 weeks cumul	12 362.	17 092.2	{2373., 77 934.}	132.347	0.475845	newly 50 weeks cumul	14 680.	21 627.	{2743., 82 983.}	138.666	0.473046
20	newly 3 weeks spec	351.	2050.75	{116., 5551.}	508.356	0.716711	newly 3 weeks cumul	2657.	18 404.7	{1619., 56 107.}	608.695	0.662328	newly 5 weeks spec	80.	530.02	{8., 1232.}	592.325	0.803406	newly 5 weeks cumul	2903.	19 907.4	{1691., 58 116.}	601.488	0.67258	newly 55 weeks cumul	3010.	20 964.9	{1964., 58 393.}	610.993	0.684809
21	newly 3 weeks spec	1266.	1792.41	{163., 6819.}	142.527	0.600858	newly 3 weeks cumul	6452.	8729.97	{1440., 27 079.}	116.574	0.448125	newly 5 weeks spec	504.	741.13	{12., 3393.}	164.617	0.832626	newly 5 weeks cumul	7741.	10 635.4	{1906., 35 054.}	123.007	0.476213	newly 52 weeks cumul	8578.	11 934.8	{2458., 42 066.}	127.251	0.496201
22	newly 3 weeks spec	1097.	1291.28	{87., 2198.}	74.4631	0.387905	newly 3 weeks cumul	5662.	21 175.6	{1816., 53 350.}	299.038	0.525329	newly 5 weeks spec	414.	118.7	{3., 321.}	76.5411	0.953273	newly 5 weeks cumul	6768.	21 670.7	{1859., 53 550.}	247.368	0.485959	newly 53 weeks cumul	7400.	21 739.3	{1859., 53 550.}	222.144	0.463923
23	newly 3 weeks spec	2274.	2646.44	{39., 5526.}	120.567	0.742298	newly 3 weeks cumul	9195.	20 777.3	{1533., 58 587.}	175.016	0.456885	newly 5 weeks spec	1192.	924.47	{0., 1022.}	121.353	1.38679	newly 5 weeks cumul	12 094.	23 233.4	{1899., 64 335.}	148.383	0.446535	newly 46 weeks cumul	15 344.	24 718.3	{2191., 64 917.}	126.824	0.451452
24	newly 3 weeks spec	2818.	1991.92	{194., 4887.}	69.1505	0.536003	newly 3 weeks cumul	11 319.	11 552.5	{2251., 31 801.}	77.6648	0.39532	newly 5 weeks spec	1477.	572.59	{25., 1363.}	69.8274	0.788084	newly 5 weeks cumul	14 831.	13 170.5	{2767., 34 626.}	71.7298	0.41645	newly 46 weeks cumul	19 249.	14 106.7	{2780., 35 270.}	65.0422	0.445259
25	newly 3 weeks spec	1110.	1052.19	{85., 2764.}	73.3486	0.430126	newly 3 weeks cumul	5663.	12 642.	{1309., 14 322.}	175.352	0.367155	newly 5 weeks spec	432.	272.05	{9., 624.}	81.9745	0.730969	newly 5 weeks cumul	6746.	13 408.6	{1394., 18 093.}	154.402	0.367117	newly 49 weeks cumul	7332.	13 892.7	{1416., 21 699.}	146.403	0.367547
26	newly 3 weeks spec	2919.	2103.84	{109., 6635.}	123.941	0.9425	newly 3 weeks cumul	11 387.	9599.29	{1866., 16 143.}	109.618	0.600869	newly 5 weeks spec	1758.	646.9	{6., 2367.}	104.597	1.42438	newly 5 weeks cumul	15 461.	11 427.5	{1958., 33 913.}	109.302	0.683639	newly 47 weeks cumul	21 236.	12 454.9	{1972., 59 928.}	104.095	0.782541
27	newly 3 weeks spec	2411.	1211.22	{0., 2078.}	108.685	2.2301	newly 3 weeks cumul	9503.	7863.27	{139., 13 572.}	123.497	0.933281	newly 5 weeks spec	1418.	293.84	{0., 1016.}	90.0226	2.52855	newly 5 weeks cumul	12 786.	8731.2	{157., 14 427.}	112.586	0.990711	newly 48 weeks cumul	17 179.	9575.92	{159., 14 430.}	100.263	1.04675
28	newly 3 weeks spec	2066.	876.82	{40., 1378.}	89.0174	0.950968	newly 3 weeks cumul	9289.	11 990.5	{1929., 25 928.}	83.0904	0.342723	newly 5 weeks spec	1123.	103.51	{0., 199.}	96.7809	1.90684	newly 5 weeks cumul	12 043.	12 389.6	{1935., 26 118.}	79.1917	0.405054	newly 52 weeks cumul	14 773.	12 446.5	{1936., 26 131.}	77.9849	0.466463
29	newly 3 weeks spec	2511.	950.66	{56., 1923.}	90.7519	0.978796	newly 3 weeks cumul	10 166.	16 125.7	{1929., 58 138.}	121.663	0.393602	newly 5 weeks spec	1397.	80.99	{1., 333.}	94.2026	2.06777	newly 5 weeks cumul	13 376.	16 471.1	{2470., 58 176.}	106.078	0.450869	newly 46 weeks cumul	17 318.	16 520.4	{3290., 58 176.}	94.5487	0.511249
30	newly 3 weeks spec	1594.	1664.7	{0., 2318.}	146.419	1.79041	newly 3 weeks cumul	7832.	14 382.3	{2222., 19 190.}	136.003	0.325361	newly 5 weeks spec	651.	971.24	{0., 1366.}	199.853	2.60976	newly 5 weeks cumul	9505.	16 580.2	{2225., 31 371.}	136.835	0.36123	newly 55 weeks cumul	10 576.	20 590.5	{2225., 82 535.}	159.563	0.392516
31	newly 3 weeks spec	771.	800.26	{105., 1242.}	70.9935	0.335592	newly 3 weeks cumul	4574.	5480.77	{1122., 7182.}	68.4873	0.246925	newly 5 weeks spec	189.	302.26	{14., 506.}	124.18	0.505459	newly 5 weeks cumul	5139.	6257.9	{1521., 7407.}	70.4359	0.248221	newly 56 weeks cumul	5392.	7412.71	{1753., 9810.}	83.5399	0.25031
32	newly 3 weeks spec	858.	1898.87	{18., 2373.}	219.784	0.694322	newly 3 weeks cumul	4649.	12 816.	{872., 50 581.}	246.224	0.524974	newly 5 weeks spec	288.	531.15	{0., 536.}	210.101	1.08906	newly 5 weeks cumul	5438.	14 370.2	{913., 61 532.}	238.839	0.536995	newly 53 weeks cumul	5832.	14 930.9	{1033., 61 545.}	232.817	0.542254
33	newly 3 weeks spec	3068.	883.34	{52., 1055.}	99.6975	1.1902	newly 3 weeks cumul	12 287.	3193.67	{674., 2592.}	95.4208	1.02132	newly 5 weeks spec	1670.	631.49	{4., 1425.}	105.185	1.43899	newly 5 weeks cumul	16 122.	4572.92	{850., 5234.}	96.8477	1.05287	newly 51 weeks cumul	20 713.	7402.23	{859., 23 797.}	98.4283	1.08165
34	newly 3 weeks spec	2614.	3330.04	{46., 12 585.}	149.386	1.01401	newly 3 weeks cumul	10 179.	27 170.4	{1367., 79 171.}	205.06	0.468588	newly 5 weeks spec	1528.	495.57	{0., 1308.}	95.9915	1.89315	newly 5 weeks cumul	13 712.	28 897.5	{1415., 80 496.}	159.446	0.455417	newly 46 weeks cumul	18 212.	29 299.7	{1415., 80 568.}	125.568	0.473791
35	newly 3 weeks spec	2810.	3029.75	{86., 12 551.}	153.307	1.01704	newly 3 weeks cumul	11 877.	11 264.9	{608., 48 397.}	122.384	0.780498	newly 5 weeks spec	1428.	1345.8	{15., 6283.}	150.109	1.32287	newly 5 weeks cumul	15 254.	14 725.2	{740., 54 595.}	129.087	0.83426	newly 51 weeks cumul	18 586.	16 770.7	{795., 88 137.}	126.319	0.879377
36	newly 3 weeks spec	2377.	1924.26	{172., 5305.}	91.639	0.56694	newly 3 weeks cumul	9678.	10 389.3	{1802., 22 599.}	87.1334	0.398593	newly 5 weeks spec	1295.	707.24	{7., 2188.}	89.271	0.865302	newly 5 weeks cumul	12 751.	12 234.	{2309., 30 107.}	84.9967	0.426837	newly 49 weeks cumul	16 300.	14 004.9	{2836., 35 875.}	83.6325	0.461715
37	newly 3 weeks spec	918.	1273.85	{0., 4576.}	153.744	2.10638	newly 3 weeks cumul	5030.	17 073.4	{1908., 46 191.}	266.704	0.442493	newly 5 weeks spec	306.	511.36	{0., 876.}	184.856	1.95875	newly 5 weeks cumul	5864.	18 392.4	{2455., 48 731.}	240.858	0.430644	newly 54 weeks cumul	6382.	19 773.3	{2532., 49 143.}	236.597	0.44847
38	newly 3 weeks spec	286.	1500.36	{0., 2450.}	524.839	2.13182	newly 3 weeks cumul	2079.	20 245.6	{4153., 74 166.}	877.173	0.73816	newly 5 weeks spec	76.	604.26	{0., 1001.}	795.632	1.9258	newly 5 weeks cumul	2297.	21 773.3	{5390., 81 404.}	851.246	0.741521	newly 54 weeks cumul	2347.	23 297.4	{5655., 81 404.}	895.756	0.775016
39	newly 3 weeks spec	1569.	2799.21	{0., 6066.}	217.284	1.68072	newly 3 weeks cumul	6961.	18 019.1	{742., 70 658.}	229.147	0.608967	newly 5 weeks spec	719.	1298.95	{0., 1144.}	224.33	2.25149	newly 5 weeks cumul	8738.	21 219.5	{749., 88 647.}	216.518	0.60798	newly 48 weeks cumul	10 021.	24 476.1	{749., 88 647.}	216.741	0.605297
40	newly 3 weeks spec	2069.	817.56	{16., 954.}	106.801	1.32536	newly 3 weeks cumul	8421.	6681.72	{626., 10 722.}	104.274	0.663693	newly 5 weeks spec	1045.	69.78	{0., 157.}	94.4919	2.40368	newly 5 weeks cumul	10 908.	6986.93	{628., 10 896.}	97.8386	0.734813	newly 49 weeks cumul	13 560.	7023.06	{631., 10 918.}	94.9494	0.811208
41	newly 3 weeks spec	1243.	785.32	{0., 738.}	124.529	2.50494	newly 3 weeks cumul	5945.	11 890.9	{1035., 47 832.}	202.015	0.566727	newly 5 weeks spec	608.	385.02	{0., 501.}	120.977	2.48455	newly 5 weeks cumul	7464.	12 814.3	{1263., 66 087.}	180.039	0.598432	newly 50 weeks cumul	8541.	15 232.2	{1263., 80 409.}	183.492	0.604715
42	newly 3 weeks spec	457.	2587.25	{143., 10 246.}	497.039	0.611308	newly 3 weeks cumul	2671.	11 924.	{690., 26 516.}	380.671	0.580152	newly 5 weeks spec	82.	1256.	{41., 2674.}	1449.44	0.820365	newly 5 weeks cumul	2987.	14 967.9	{850., 48 755.}	433.58	0.601853	newly 54 weeks cumul	3092.	17 798.5	{978., 72 985.}	506.096	0.623736
43	newly 3 weeks spec	2726.	2521.66	{720., 6734.}	83.7579	0.483146	newly 3 weeks cumul	11 008.	26 332.1	{10 117., 60 571.}	144.662	0.277807	newly 5 weeks spec	1377.	325.66	{32., 806.}	79.7894	0.956529	newly 5 weeks cumul	14 359.	27 553.4	{10 483., 63 104.}	113.578	0.264519	newly 47 weeks cumul	18 000.	27 755.3	{10 540., 63 415.}	95.1147	0.282431
44	newly 3 weeks spec	1026.	3577.39	{206., 14 308.}	280.831	0.455175	newly 3 weeks cumul	5335.	14 817.	{1520., 47 754.}	213.528	0.416513	newly 5 weeks spec	393.	1860.28	{78., 4519.}	405.822	0.536749	newly 5 weeks cumul	6350.	19 245.4	{1915., 66 675.}	235.916	0.426365	newly 53 weeks cumul	7041.	24 327.8	{2036., 93 556.}	273.255	0.440642
45	newly 3 weeks spec	374.	795.55	{22., 2254.}	187.992	0.653203	newly 3 weeks cumul	2546.	14 088.	{888., 34 416.}	472.671	0.649692	newly 5 weeks spec	135.	88.42	{0., 214.}	93.9111	1.36357	newly 5 weeks cumul	2913.	14 432.2	{890., 36 398.}	415.555	0.614751	newly 52 weeks cumul	3058.	14 484.5	{890., 37 214.}	394.033	0.600451
46	newly 3 weeks spec	1322.	1303.13	{54., 3625.}	108.222	0.680519	newly 3 weeks cumul	7028.	6508.81	{455., 16 319.}	94.7258	0.541724	newly 5 weeks spec	520.	528.93	{8., 2076.}	118.744	0.838659	newly 5 weeks cumul	8416.	7870.22	{504., 20 353.}	97.346	0.564128	newly 52 weeks cumul	9218.	8901.71	{537., 24 783.}	100.825	0.580265
47	newly 3 weeks spec	877.	3150.69	{570., 6939.}	276.637	0.395094	newly 3 weeks cumul	5116.	14 710.2	{2511., 55 218.}	211.265	0.391088	newly 5 weeks spec	280.	1509.29	{143., 3734.}	465.082	0.497437	newly 5 weeks cumul	5861.	18 411.6	{3441., 61 824.}	233.688	0.398411	newly 54 weeks cumul	6385.	23 229.9	{4312., 63 341.}	280.482	0.43006
48	newly 3 weeks spec	1336.	1381.19	{0., 2939.}	113.061	2.13458	newly 3 weeks cumul	7014.	25 271.3	{2496., 84 903.}	287.276	0.450709	newly 5 weeks spec	498.	633.84	{0., 1471.}	132.582	1.98753	newly 5 weeks cumul	8264.	26 798.1	{3784., 84 903.}	248.485	0.426036	newly 54 weeks cumul	8948.	30 182.4	{4553., 84 903.}	257.553	0.454962
49	newly 3 weeks spec	215.	677.94	{54., 1062.}	245.721	0.431218	newly 3 weeks cumul	1583.	5610.69	{1310., 5820.}	268.938	0.378399	newly 5 weeks spec	43.	169.14	{2., 384.}	332.326	0.634106	newly 5 weeks cumul	1735.	6102.74	{1581., 6380.}	265.584	0.381041	newly 56 weeks cumul	1768.	6435.08	{1581., 7760.}	276.879	0.396364
50	newly 3 weeks spec	2185.	4087.89	{27., 15 308.}	200.38	0.999713	newly 3 weeks cumul	9176.	27 717.8	{1801., 73 784.}	271.184	0.560333	newly 5 weeks spec	1236.	1000.25	{1., 3173.}	128.135	1.7324	newly 5 weeks cumul	12 158.	30 761.	{1822., 85 992.}	232.733	0.608383	newly 47 weeks cumul	15 907.	31 550.9	{1822., 87 601.}	186.831	0.636366
